# Why and how to optimize glucocorticoid treatment in rheumatoid arthritis

**DOI:** 10.1186/ar3726

**Published:** 2012-03-08

**Authors:** Maurizio Cutolo

**Affiliations:** 1 Research Laboratory and Academic Unit of Clinical Rheumatology, Department of Internal Medicine, University of Genova, Genova, Italy

## 

Glucocorticoids (GC) are the most potent anti-inflammatory and immunosuppressive hormones mainly produced by the adrenal glands in humans.

The central nervous clock system, under the influence of light/dark alternation, "creates" the internal circadian rhythms and the organisms by "feeling" these rhythmic external changes, synchronize their physical activities, including sleep, related nocturnal hormone synthesis and immune function [1].

As a matter of fact, GC rise during the night around 3 am and start to exert anti-inflammatory and immunosuppressive activities. On the other hand, the nocturnal pineal hormone melatonin, that rises earlier in the night with darkness, has been linked to chronic inflammation since at normal to slightly elevated concentrations stimulate many aspects of the immune/inflammatory response, especially at the level of macrophages [2].

The immune-supportive role of melatonin and the reduced immune suppression linked to decreased endogenous GC (due to the chronic stress of the disease) have been delineated in the context of the circadian rhythms of immune/inflammatory reaction and related clinical (morning) symptoms, at least in RA [3].

Therefore, the most advanced approach to optimize the risk-benefit ratio of long-term low-dose GC treatment is the GC-chronotherapy, using a modified release (MR) prednisone (release during the night) and following the circadian rhythms.

In fact in RA, the circadian rhythms of clinical symptoms are more evident in the early morning hours, since preceded by nocturnal elevated levels of pro-inflammatory cytokines (i.e.IL-6). Therefore, since prevention of nocturnal rise of pro-inflammatory cytokines by GC therapy would be more effective than treating established symptoms in the morning and might reduce doses and side effects, a MR prednisone was developed, which releases prednisone approximately four hours after ingestion (i.e., at approximately 2-3 am when taken at bedtime) [4]. In addition to all recognized therapeutic effects obtained with conventional prednisone, MR prednisone was shown to have similar profile of adverse effects but without additional suppression of hypothalamic-pituitary-adrenal (HPA) axis [5].

In conclusion, night-time low dose long-term GC therapy in chronic rheumatic diseases such as RA, is today considered as an "hormonal replacement therapy" that optimally implement the peripheral insufficiency of endogenous GC in modulating the immune/inflammatory reaction.
